# Global, regional, and national burden of childhood cardiovascular disease: trends from 1990 to 2021

**DOI:** 10.3389/fped.2024.1495238

**Published:** 2024-12-11

**Authors:** Mingling Wang, Junling Yi, Zuolei Chen

**Affiliations:** ^1^ Department of Anesthesiology, Qingdao Women and Children’s Hospital, School of Medicine, Shandong University, Jinan, China; ^2^ Central Laboratory of Prenatal Diagnosis and Obstetrics, Qingdao Municipal Hospital, Qingdao, China; ^3^ Department of Anesthesiology of the Affiliated Hospital of Qingdao Binhai University, Qingdao, China

**Keywords:** childhood, metabolic disease, cardiovascular disease, prevalance, death

## Abstract

**Background:**

Childhood cardiovascular disease (CVD) is an emerging public health concern, with rising incidence linked to obesity and diabetes. Despite advancements in care, significant disparities persist across regions and socioeconomic groups. This study analyzed the global, regional, and national burden of childhood CVD from 1990 to 2021.

**Methods:**

A cross-sectional study utilizing data from the Global Burden of Disease (GBD) 2021 was conducted. We analyzed children aged 0–14 years, categorizing them into age groups and assessing trends in incidence, mortality, and disability-adjusted life years (DALYs) across 204 countries. Statistical analyses included linear regression to calculate estimated annual percentage changes and assess temporal trends.

**Results:**

The study revealed a 25% global increase in childhood CVD incidence over three decades, with significant disparities observed across different socioeconomic regions. Mortality and DALYs associated with CVD had decreased globally by 59% and 55% respectively, reflecting advances in medical technology and healthcare access. However, these improvements were not uniformly distributed, with low socio-demographic index regions exhibiting both the highest incidences and slowest declines in CVD-related health burdens. Environmental risks, such as extreme temperatures, also contributed to CVD mortality.

**Conclusions:**

While mortality and DALYs due to childhood CVD have declined globally, rising incidence and persistent disparities highlight the need for targeted interventions. Addressing socio-economic factors and enhancing access to quality care are crucial for reducing the global burden of childhood CVD.

## 1 Introduction

Childhood cardiovascular disease (CVD) is increasingly recognized as a significant public health issue, affecting morbidity and mortality from an early age (1). Although traditionally associated with adults, the rise of CVD in children underscores the urgent need for specialized research in this demographic, especially as these conditions can influence lifelong health trajectories. The increasing prevalence of obesity, diabetes, and other metabolic disorders among children has led to a concerning trend towards earlier onset of traditionally adult diseases, underscoring the importance of targeted pediatric research (2–5).

Existing studies from high-income countries provide some insights into the prevalence, risk factors, and outcomes of childhood CVD (6, 7). However, a comprehensive global perspective remains limited. This gap in research highlights a profound disparity in our understanding of childhood CVD across different socioeconomic landscapes and emphasizes the need for more inclusive research efforts.

Despite advancements in medical care and technology, which have improved the diagnosis and management of childhood CVD, there remains a critical need for updated and detailed assessments of the global burden of these diseases. Notably, variations in healthcare access, quality of care, and public health initiatives across regions contribute to differing outcomes, underscoring the importance of examining the burden of childhood CVD at national, regional, and global levels (8–10). Moreover, understanding the temporal trends in childhood CVD is crucial for identifying shifts in disease patterns and the effectiveness of interventions over time.

This study aimed to address critical knowledge gaps by offering a comprehensive analysis of the global, regional, and national burden of childhood CVD from 1990 to 2021. Through the use of extensive data sources and rigorous epidemiological methods, the study will elucidate trends in incidence, mortality, and disability-adjusted life years (DALYs) related to childhood CVD across various regions and income levels. The findings are expected to inform public health policies, guide resource allocation, and ultimately contribute to global efforts in reducing the burden of childhood CVD.

## 2 Methods

### 2.1 Study design and data sources

This cross-sectional study analyzed children aged 0–14 years with CVD, adhering to the STROBE guidelines. Data were extracted from the Global Health Data Exchange, developed by collaborators of the Global Burden of Disease (GBD) 2021 study (11). This comprehensive database assessed the epidemiologic trends of various diseases across 204 countries from 1990 to 2021. We focused on the age-specific burden of CVD in children, categorizing them into three age groups: under 5 years, 5–9 years, and 10–14 years. We analyzed the incidence, mortality, and DALYs alongside their corresponding global, regional, and national trends. Additionally, we calculated the mean estimated annual percentage changes (EAPCs) using linear regression to assess temporal trends. EAPC is derived from the regression of the natural logarithm of rates over time, with positive values indicating increasing trends and negative values indicating declining trends, reflecting the annual rate of change.

### 2.2 Sociodemographic index (SDI)

The Socio-Demographic Index (SDI) measures a region's development based on fertility rates, education levels, and per capita income, with higher SDI values indicating greater socioeconomic development (12). This study categorizes countries into five SDI levels-low, low-middle, middle, high-middle, and high—to explore the correlation between childhood CVD burden and socioeconomic development, examining how disease incidence and mortality vary across these levels.

### 2.3 Statistical analysis

We primarily focused on the rates of incidence, mortality, and DALYs per 100,000 population to describe the burden of childhood CVD, presenting these with 95% uncertainty intervals (UI). To determine temporal trends, we calculated EAPCs and their 95% confidence intervals (CI) using linear modeling. A negative upper limit of an EAPC and its confidence interval suggests a declining trend in rates, whereas a positive lower limit indicates an increasing trend. The impact of global risk factors on childhood CVD mortality was also assessed. All statistical analyses were performed using R-Studio V4.1.2, with significance set at *P* < .05.

## 3 Results

### 3.1 Global trends

#### 3.1.1 Incidence

**Figure 1:**
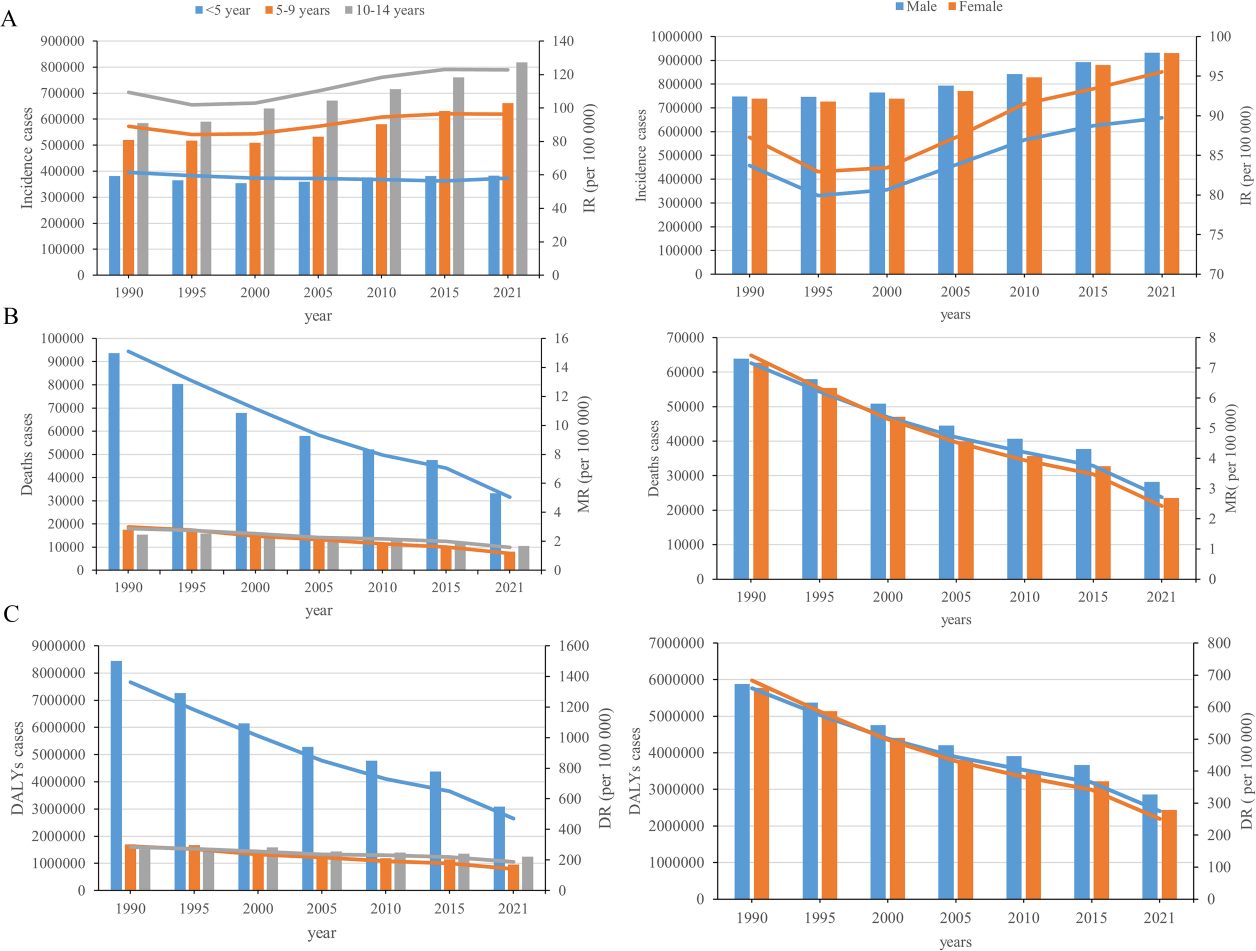
Trends in childhood cardiovascular disease incidence, deaths, and disability-adjusted life years, by Sex-age stratification From 1990 to 2021. **(A)** Incidence; **(B)** deaths; **(C)** DALYs.

In 2021, the global incidence of CVD in children was 1,861,693 cases (95% UI, 1,335,751–2,531,860). From 1990 to 2021, the global incidence cases of childhood CVD increased by 25%. The corresponding incidence rate (IR) increased from 85.45 (95% UI, 64.12 −112.67) per 100,000 people in 1990 to 92.54 (95% UI, 66.39–125.85) per 100,000 people in 2021, with an EAPC of 0.43 (95% CI, 0.36 to 0.5). From 1990 to 2021, the IR of childhood CVD in children under 5 years of age showed a decreasing trend, while the IR in children aged 10–14 years showed a continuous increasing trend. The fastest increase in CVD incidence was observed in children aged 10–14 years, with a 40% rise. The highest IR, 122.72 (95% UI, 74.38–188.76), was found in the age group of 10–14 years. In 2021, the IR of CVD was higher in females than in males [males: 89.73 (95% UI, 65.07–120.96); females: 95.53 (95% UI, 68.02–131.10)]. This pattern was consistent across all age groups, with the highest IR observed in children aged 10–14 years [males: 116.77 (95% UI, 71.50–178.46); females: 129.05 (95% UI, 76.56–200.14)] (Figure 1A and Supplementary Table S1).

#### 3.1.2 Deaths

In 2021, there were 51,728 (95% UI, 43,452–60,332) CVD deaths in children worldwide. From 1990 to 2021, global childhood CVD deaths cases decreased by 59.15%. The corresponding mortality rate (MR) decreased from 7.28 (95% UI, 6.34–8.71) in 1990 to 2.57 (95% UI, 2.16–3.00) in 2021, with an EAPC of −2.97 (95% CI, −3.09 to −2.85). From 1990 to 2021, there was a downward trend in the MR of CVD among children of all ages. The greatest decline in both CVD deaths and MR was observed in children under 5 years of age. This age group also had the highest mortality rate of 5.04 (95% UI, 4.06–6.09). In 2021, the MR of CVD was higher in males than in females [males: 2.71 (95% UI, 2.24–3.19); females: 2.42 (95% UI, 2.06–2.81)]. Across all age groups, the MR of CVD was higher in males than in females for children younger than nine years, and higher in females than in males for those aged 10–14 years. (Figure 1B and Supplementary Table S2).

#### 3.1.3 DALYs

In 2021, there were 5,300,050 (95% UI, 4,579,016–6,083,866) global DALY cases of CVD in children. From 1990 to 2021, there was a 55% (95% UI, 48%−61%) decline in the global number of DALY cases of childhood CVD. The corresponding DALY rate (DR) decreased from 670.69 (95% UI, 594.14–791.76) in 1990 to 263.44 (95% UI, 227.60–302.40) in 2021, with an EAPC of −2.68 (95% CI, −2.78 to −2.58). From 1990 to 2021, the number and rate of DALYs declined among children of all ages, with the largest decrease observed in children younger than 5 years of age. The highest rate of DALYs in 2021 was 469.57 (95% UI, 383.95–562.20) in the age group younger than 5 years. In 2021, sex-specific DALY rates for CVD were higher in males than in females [males: 275.29 (95% UI, 234.88–318.73); females: 250.81 (95% UI, 216.84–288.11)]. Across all age groups, DALY rates for CVD were higher in males than in females for children under 9 years of age, while in the 10–14 years age group, DALY rates were higher for females than for males (Figure 1C and Supplementary Table S3).

### 3.2 SDI regional trends

#### 3.2.1 Incidence

**Figure 2:**
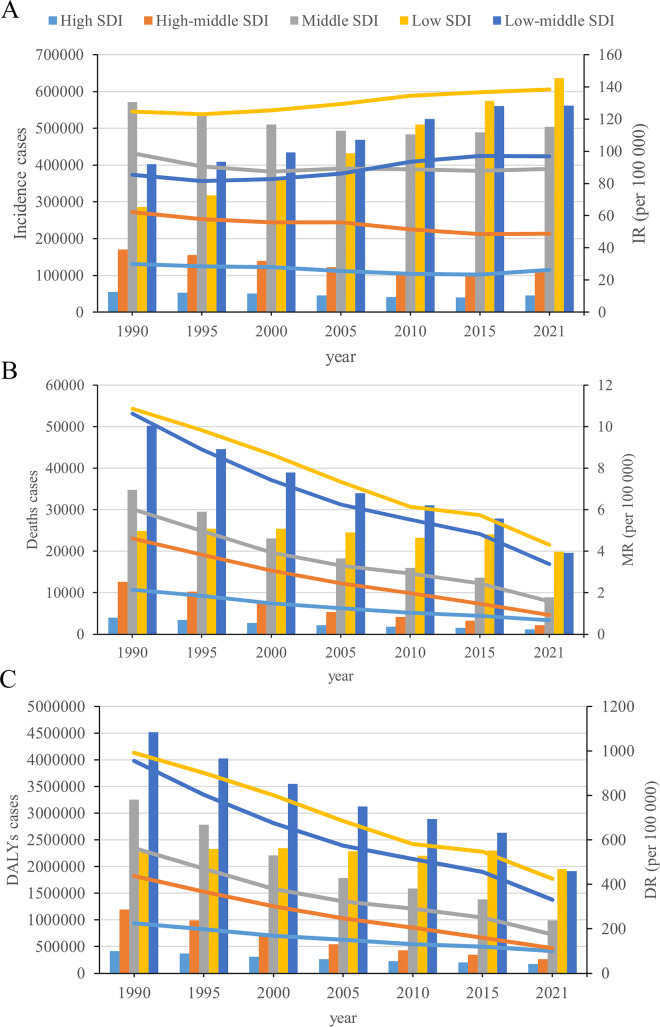
Epidemiologic trends of incidence, death, and DALYs rates in 5 SDI regions for childhood cardiovascular disease from 1990 to 2021. **(A)** Incidence; **(B)** deaths; **(C)** DALYs.

The low SDI region had the highest number of childhood CVD cases in 2021 (636,598; 95% UI, 442,425–876,582). The incident cases in the low SDI region increased by 123%, although it was noted that the incident cases in the high-middle SDI region decreased by 34%. The greatest increase in the incidence of childhood CVD occurred in the low-middle SDI region (EAPC, 0.66; 95% CI, 0.56–0.77) (Figure 2A and Supplementary Table S1).

#### 3.2.2 Deaths

From 1990 to 2021, all 5 SDI regions exhibited a decrease in the number of CVD-associated deaths. The deaths cases in the high-middle SDI region decreased by 83%. The low SDI region also had the highest number of CVD-associated deaths in 2021 (19,854; 95% UI, 15,376–24,684). In 2021, the childhood CVD-associated mortality rate was highest in the low SDI region (4.31; 95% UI, 3.34–5.36) and lowest in the high SDI region (0.68; 95% UI, 0.62–0.72). The high-middle SDI region had the lowest EAPC in the childhood CVD-associated mortality rate (−4.83; 95% UI, −5.02 to −4.65) (Figure 2B and Supplementary Table S2).

#### 3.2.3 DALYs

In 2021, the low SDI region had the highest number of CVD-associated DALYs (1,953,670; 95% UI, 1,560,955–2,377,202). The high-middle SDI region experienced the greatest decrease (78%) in the number of CVD-associated DALYs. The low SDI region had the highest rate of CVD-associated DALYs at 424.50 (95% UI, 339.17–516.53). The high-middle SDI region had the greatest decrease in the rate of DALYs, corresponding to an EAPC of −4.15 (95% CI, −4.26 to −4.05) (Figure 2C and Supplementary Table S3).

### 3.3 Geographic regional trends

#### 3.3.1 Incidence

**Figure 3:**
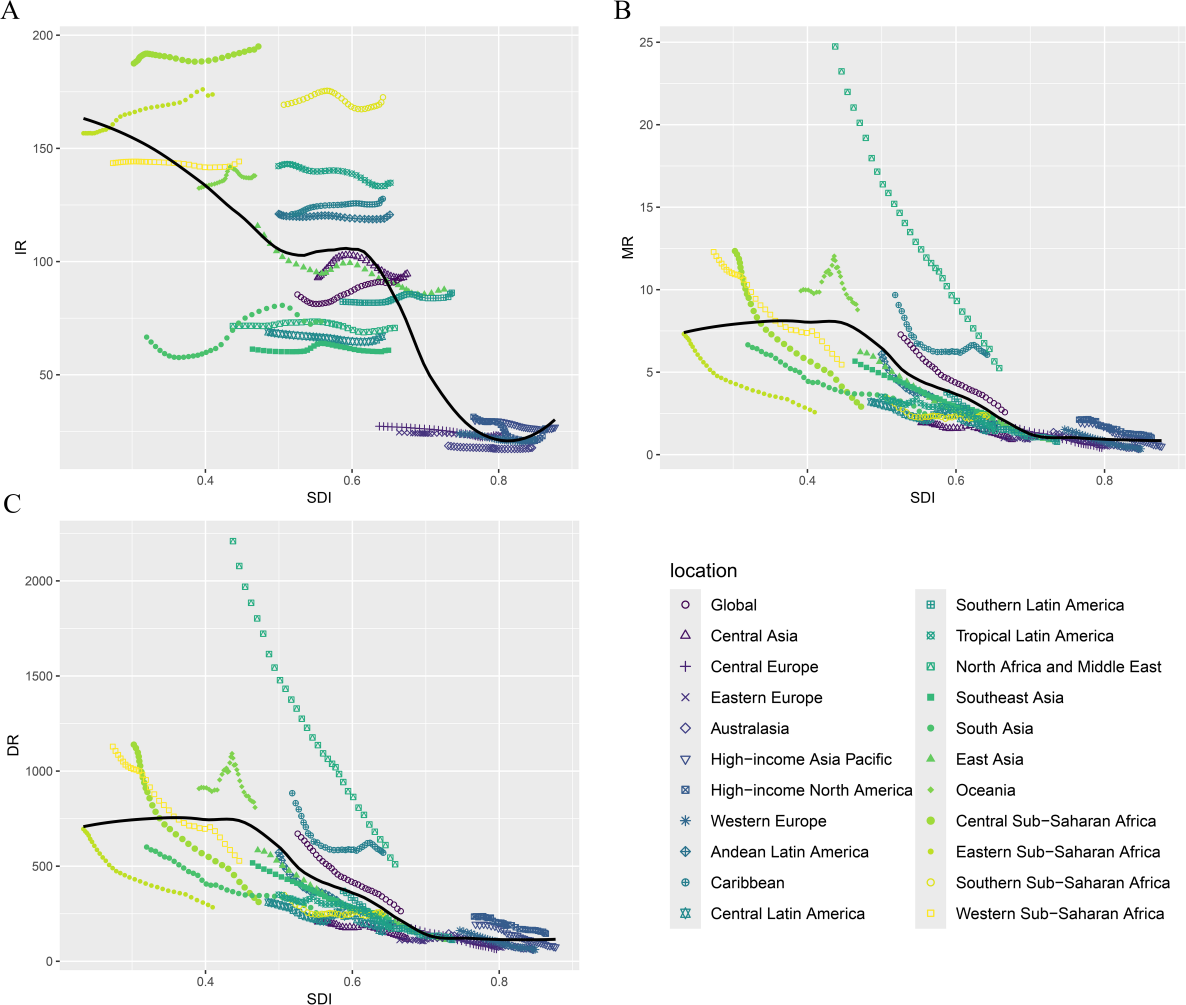
Incidence, death, and DALYs rates for childhood cardiovascular disease by SDI from 1990 to 2021. **(A)** Incidence rate; **(B)** death rate; **(C)** DALYs Rate.

Among the 21 geographic regions, South Asia had the most CVD cases in 2021 (372,929 cases; 95% UI, 253,626–517,465 cases), whereas Australasia had the fewest (1,018 cases; 95% UI, 811–1,266 cases). Central Sub-Saharan Africa had the highest incidence of CVD in children (195.01; 95% UI, 132.94–276.60), while Australasia had the lowest incidence of pediatric CVD (17.76; 95% UI, 14.15–22.10). From 1990 to 2021, South Asia had the largest increase in childhood CVD incidence (EAPC, 1.04; 95% CI, 0.75 to 1.32), while High-income North America had the largest decline (EAPC, −1.31; 95% CI, −1.63 to −0.99). In 2021, Central Sub-Saharan Africa (SDI, 0.47) had the highest incidence of childhood CVD, whereas Australasia (SDI, 0.85) had the lowest incidence. The global SDI in 2021 was 0.67; nine regions (e.g., Central Sub-Saharan Africa and Eastern Sub-Saharan Africa) had a higher incidence of childhood CVD than the global average, while 12 regions (e.g., Western Europe and Australasia) had a lower incidence than the global average (92.54) (Figure 3A and Supplementary Table S1).

#### 3.3.2 Deaths

In 2021, South Asia had the highest number of pediatric CVD-related deaths (12,570; 95% UI, 10,320–15,017). Oceania had the highest pediatric CVD-related mortality rate (8.78; 95% UI, 6.69–11.13). Oceania had the smallest decrease in CVD-related mortality (EAPC, −0.21; 95% CI, −0.52 to 0.1), while East Asia had the largest decrease (EAPC, −5.98; 95% CI, −6.17 to −5.78). In 2021, Oceania (SDI, 0.47) had the highest pediatric CVD-related mortality rate, while Australasia (SDI, 0.85) had the lowest mortality rate. As noted earlier, the global SDI for 2021 was 0.67; six regions had child CVD-related mortality rates above the global average, while 15 regions had child CVD-related mortality rates below the global average (2.57) (Figure 3B and Supplementary Table S2).

#### 3.3.3 DALYs

In 2021, South Asia had the highest number of childhood CVD-related DALYs (1,226,609; 95% UI, 1,028,500–1,444,476), whereas Australasia had the lowest (3,532; 95% UI, 2,882–4,308). Oceania had the highest DALY rate (809.02; 95% UI, 626.36–1,015.14), while Western Europe had the lowest DALY rate (61.49; 95% UI, 50.14–75.56). From 1990 to 2021, Oceania had the smallest decrease in DALY rates (EAPC, −0.19; 95% CI, −0.49 to 0.11), while East Asia had the largest decrease (EAPC, −5.16; 95% CI, −5.27 to −5.05). The global SDI for 2021 was 0.67; six regions (e.g., Oceania) had DALY rates above the global average, while 15 regions (e.g., East Asia) had DALY rates below the global average (263.44) (Figure 3C and Supplementary Table S2).

### 3.4 National trends

#### 3.4.1 Incidence

**Figure 4:**
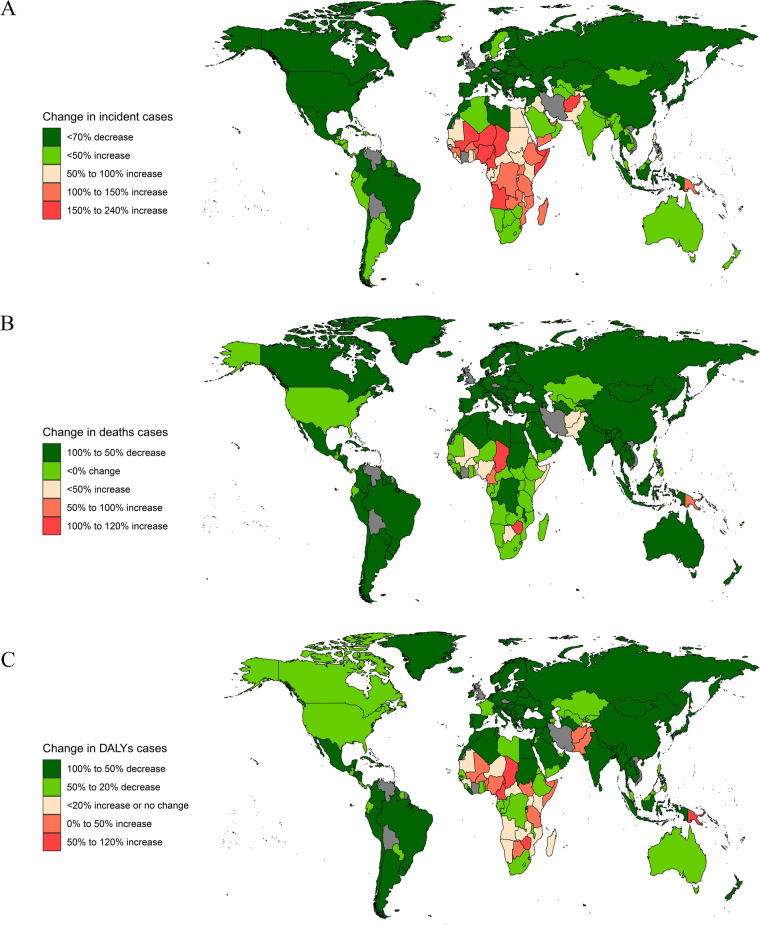
Change in incidence, deaths, and DALYs cases of diabetes in children in 204 countries and territories. **(A)** Incidence; **(B)** deaths; **(C)** DALYs.

**Figure 5:**
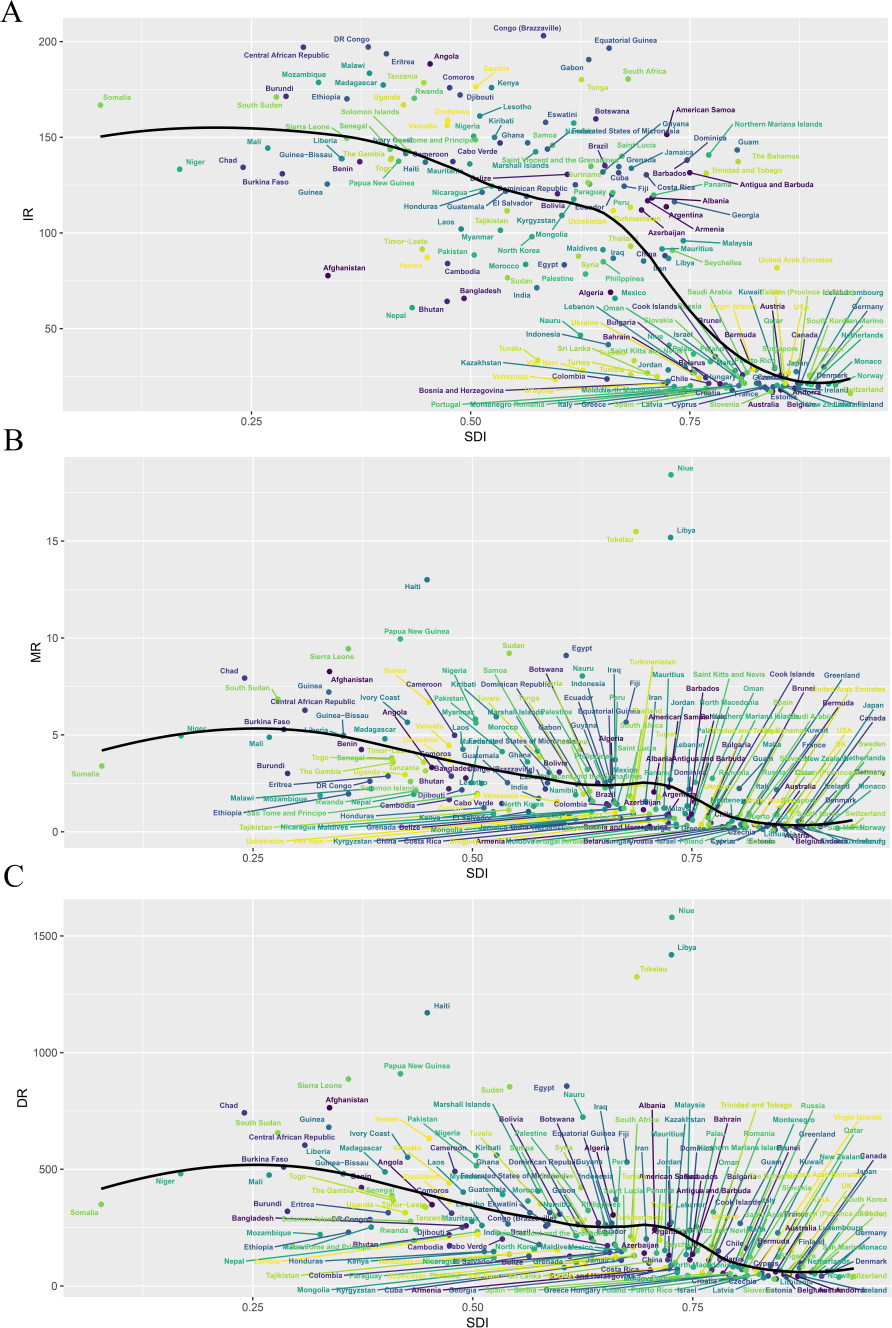
Incidence, deaths, and DALYs rates of cardiovascular disease in children in 204 countries by SDI in 2021. **(A)** Incidence rate; **(B)** death rate; **(C)** DALYs Rate.

In 2021, among 204 countries, India had the most cases of childhood CVD (261,465; 95% UI, 177,765–362,631). Congo had the highest incidence rate of childhood CVD (203.09; 95% UI, 139.05–289.53). India (EAPC, 1.28; 95% CI, 0.82–1.75) had the largest increase in childhood CVD incidence, while the USA (EAPC, −1.40; 95% CI, −1.75 to −1.06) had the largest decrease. In 2021, Congo (SDI, 0.58) had the highest incidence of childhood CVD, whereas Switzerland (SDI, 0.93) had the lowest incidence. The global incidence of childhood CVD in 2021 was 92.54; incidence rates were above the global mean in 99 countries and below the global mean in 105 countries (Figures 4A, 5A and Supplementary Figures 1A, 2A).

#### 3.4.2 Deaths

In 2021, India had the highest number of childhood CVD-associated deaths (6,320; 95% UI, 4,964–7,739). Niue had the highest childhood CVD-associated mortality rate (18.42; 95% CI, 16.24–20.81), while Slovenia had the lowest mortality rate (0.08; 95% CI, 0.07–0.09). Zimbabwe (EAPC, 3.18; 95% CI, 2.48–3.88) saw the greatest increase in the mortality rate, while Serbia (EAPC, −7.65; 95% CI, −8.16 to −7.15) had the greatest decreases. In 2021, Niue (SDI, 0.73) had the highest deaths rate of childhood CVD, whereas Slovenia (SDI, 0.84) had the lowest deaths rate. The global deaths rate of childhood CVD were above the global mean in 60 countries and below the global mean in 144 countries (Figures 4B, 5B and Supplementary Figures 1B, 2B).

#### 3.4.3 DALYs

In 2021, India had the highest number of CVD-associated childhood DALYs (650,429; 95% UI, 526,269–788,955). Niue had the highest rate of childhood CVD-associated DALYs (1,579.61; 95% UI, 1,398.22–1,779.30). Zimbabwe (EAPC, 2.45; 95% CI, 1.95–2.96) had the greatest increase in the DALY rate, while Saudi Arabia (EAPC, −6.55; 95% CI, −6.63 to −6.46) had the greatest decrease. Niue (SDI, 0.73) had the highest rate of childhood CVD-associated DALYs, while Slovenia (SDI, 0.84) had the lowest rate. The rates were above the global mean in 65 countries and below the global mean in 139 countries (Figures 4C, 5C and Supplementary Figure 1C, 2C).

### 3.5 Risk factor

In the GBD, the risk factors for CVD mortality in children were primarily environmental/occupational risks, which mainly include low temperature and high temperature, causing mortality rates of 0.05 (95% UI, 0.03–0.07) and 0.04 (95% UI, 0.01–0.07), respectively. North Africa and the Middle East had the highest mortality rate due to high temperature, at 0.12 (95% UI, 0.03–0.22). Oceania had the highest mortality rate due to low temperature, at 0.12 (95% UI, 0.07–0.17).

## 4 Discussion

The findings from this extensive analysis covering the global, regional, and national burden of childhood CVD over three decades (1990–2021) underscore the dynamic and complex landscape of pediatric health challenges that transcend traditional boundaries of age and geography. While incidence has increased, significant reductions in mortality and DALYs indicate progress in healthcare and prevention. However, these advances coexist with ongoing disparities in healthcare access and outcomes, emphasizing the need for targeted efforts to address pediatric health challenges across different regions and populations.

The results revealed a 25% increase in the global incidence of childhood CVD between 1990 and 2021. This rise was particularly pronounced in the 10–14 age group, which experienced a 40% increase in incidence, with females showing higher incidence rates than males. This trend could be attributed to a combination of factors, including the rising prevalence of obesity, diabetes, and other metabolic disorders among children, as well as increased awareness and improved diagnostic capabilities over time (13, 14). Changing lifestyle patterns, including diet and physical activity, also play a significant role, underscoring the need for targeted public health initiatives focused on prevention and early intervention in the pediatric population (15, 16). The growing burden of these risk factors in children highlights the need for early intervention and preventive measures to curb the onset of CVD in this population.

Conversely, the 59.15% reduction in mortality and 55% decrease in DALYs for childhood CVD globally reflect advances in medical technology, diagnostics, and therapeutic interventions. The rise of pediatric cardiology and global health efforts, especially in under-resourced areas, have likely contributed to these improvements (17, 18). However, persistent regional and sex disparities indicate that access to quality care remains uneven. The largest reduction in mortality was observed in children under five years old, which is encouraging as this age group is particularly vulnerable to severe outcomes. However, the mortality rate remains higher in males than in females for children under 9 years old, while in the 10–14 years age group, females had a higher mortality rate. These findings underscore the importance of sex-specific approaches in managing and preventing childhood CVD.

The regional analysis reveals significant disparities in childhood CVD between high- and low-SDI regions, with the latter having the highest incidence, mortality, and DALY rates in 2021 and the greatest increase in CVD incidence over time. Low-SDI regions often face limited access to healthcare, poor-quality care, and a lack of public health policies targeting CVD prevention in children (19–21). In contrast, high-middle SDI regions saw the greatest reductions in mortality and DALY rates, benefiting from better healthcare infrastructure and public health interventions (22). The disproportionate burden of childhood CVD in low-SDI regions underscores the impact of socio-economic factors on health outcomes. Low education levels contribute to poor health literacy, delayed diagnosis, and reduced early intervention, leading to worse CVD outcomes. Inadequate healthcare infrastructure and economic constraints further exacerbate these issues, with limited access to trained professionals, diagnostic services, and preventive care. Significant differences in access to advanced medical technologies between high- and low-SDI regions highlight the need for targeted investments in healthcare systems and prevention programs in resource-limited areas to reduce childhood CVD mortality and morbidity.

Geographic and national trends in childhood CVD reveal significant disparities. In 2021, South Asia had the highest number of childhood CVD cases, while Central Sub-Saharan Africa recorded the highest incidence rate. These high rates underscore the need for region-specific strategies that address both social determinants of health and direct CVD risk factors. In contrast, High-income North America saw the largest decline in CVD incidence, highlighting the effectiveness of preventive measures in that region. Mortality and DALY rates varied, with Oceania highest and Western Europe lowest, reflecting disparities in healthcare quality. Nationally, India bore the heaviest CVD burden, while Congo and Niue had high incidence and mortality rates. The USA saw the largest decline in incidence, offering valuable insights for global health initiatives. Tailored interventions are essential to address the unique challenges each country faces in managing childhood CVD.

The study identified extreme temperatures as significant environmental contributors to childhood CVD mortality. North Africa and the Middle East had the highest mortality due to heat, while Oceania had the highest mortality due to cold. These findings emphasize the vulnerability of children to extreme temperatures, which can trigger dehydration, electrolyte imbalances, or hypothermia, especially in those with pre-existing CVD (23, 24). Socio-economic factors, such as inadequate housing and limited healthcare access, amplify these risks. Addressing these environmental factors requires targeted public health interventions, such as improving housing, access to heating or cooling systems, and early warning systems for extreme weather events. Climate adaptation strategies are crucial to reducing childhood CVD mortality in regions facing extreme temperatures.

Despite the comprehensive nature of this study, several limitations must be acknowledged. First, reliance on data from the Global Burden of Disease (GBD) study may introduce potential biases, particularly regarding the accuracy and completeness of data from low-resource settings, where underreporting and variability in data quality are more prevalent. Furthermore, inconsistencies in data standardization across different countries could affect the comparability of results. Additionally, the study design precludes drawing causal inferences between socio-economic factors and childhood CVD outcomes. Changes in diagnostic criteria, reporting systems, and health-seeking behaviors over the 31-year period may have also influenced the observed trends, complicating their interpretation. Addressing these limitations highlights the need for continued improvement in global health data collection and standardization to enhance future analyses.

In conclusion, while progress has been made in reducing the global burden of childhood CVD, significant challenges remain. The disparities across regions and between sexes highlight the need for tailored, context-specific health interventions.

Effective management and prevention strategies must be underpinned by a commitment to addressing the broader socio-economic factors that contribute to health disparities. Addressing these complex challenges requires a coordinated, global effort that leverages both scientific advancements and policy-level interventions to ensure a healthier future for children worldwide.

## 5 Conclusions

This extensive analysis of the global burden of childhood CVD from 1990 to 2021 reveals critical insights into the epidemiological trends and disparities in disease impact across different regions and demographics. While there has been a noteworthy global decline in mortality and DALYs due to childhood CVD, the increase in incidence, particularly among older children, highlights the evolving nature of this public health challenge. The disparities observed across socio-demographic indices and between sexes emphasize the need for targeted interventions that address both the direct health needs and the underlying socio-economic factors contributing to these disparities. This study highlights the urgent need for evidence-based strategies that prioritize early prevention, equitable access to care, and comprehensive management of childhood cardiovascular disease. By focusing on these areas, policymakers and healthcare providers can reduce the global burden of childhood CVD and promote healthier future generations. The findings also emphasize the importance of sustained investment in pediatric cardiovascular research and inclusive public health initiatives tailored to the unique needs of children worldwide.

## Data Availability

The original contributions presented in the study are included in the article/Supplementary Material, further inquiries can be directed to the corresponding authors.

## References

[1] Salama M Balagopal B Fennoy I Kumar S. Childhood obesity, diabetes. and cardiovascular disease risk. J Clin Endocrinol Metab. (2023) 108:3051–66. 10.1210/clinem/dgad361 37319430

[2] Di Cesare M Sorić M Bovet P Miranda JJ Bhutta Z Stevens GA The epidemiological burden of obesity in childhood: a worldwide epidemic requiring urgent action. BMC Med. (2019) 17:212. 10.1186/s12916-019-1449-8 31760948 PMC6876113

[3] Zhang K Kan C Han F Zhang J Ding C Guo Z And national epidemiology of diabetes in children from 1990 to 2019. JAMA Pediatr. (2023) 177:837–46. 10.1001/jamapediatrics.2023.2029 37399036 PMC10318549

[4] Noubiap JJ Nansseu JR Lontchi-Yimagou E Nkeck JR Nyaga UF Ngouo AT Global, regional, and country estimates of metabolic syndrome burden in children and adolescents in 2020: a systematic review and modelling analysis. Lancet Child Adolesc Health. (2022) 6:158–70. 10.1016/S2352-4642(21)00374-6 35051409

[5] GBD 2021 Diabetes Collaborators. Global, regional, and national burden of diabetes from 1990 to 2021, with projections of prevalence to 2050: a systematic analysis for the global burden of disease study 2021. Lancet. (2023) 402:203–34. 10.1016/S0140-6736(23)01301-6 37356446 PMC10364581

[6] Kartiosuo N Raitakari OT Juonala M Viikari J Sinaiko AR Venn AJ Cardiovascular risk factors in childhood and adulthood and cardiovascular disease in middle age. JAMA Netw Open. (2024) 7:e2418148. 10.1001/jamanetworkopen.2024.18148 38913374 PMC11197443

[7] Wu F Jacobs DR Jr Daniels SR Kähönen M Woo JG Sinaiko AR Non-high-density lipoprotein cholesterol levels from childhood to adulthood and cardiovascular disease events. JAMA. (2024) 331:1834–44. 10.1001/jama.2024.4819 38607340 PMC11151142

[8] Kimokoti RW Hamer DH. Nutrition, health, and aging in Sub-saharan Africa. Nutr. Rev. (2008) 66:611–23. 10.1111/j.1753-4887.2008.00113.x 19019023

[9] Little MO Morley JE. Healthcare for older adults in North America: challenges, successes and opportunities. Age Ageing. (2022) 51:afac216. 10.1093/ageing/afac216 36209783

[10] Justo N Espinoza MA Ratto B Nicholson M Rosselli D Ovcinnikova O Real-world evidence in healthcare decision making: global trends and case studies from Latin America. Value Health. (2019) 22:739–49. 10.1016/j.jval.2019.01.014 31198192

[11] GBD 2021 Diseases and Injuries Collaborators. Global incidence, prevalence, years lived with disability (YLDs), disability-adjusted life-years (DALYs), and healthy life expectancy (HALE) for 371 diseases and injuries in 204 countries and territories and 811 subnational locations, 1990–2021: a systematic analysis for the global burden of disease study 2021. Lancet. (2024) 403:2133–61. 10.1016/S0140-6736(24)00757-8 38642570 PMC11122111

[12] Ahr H. Global burden of 369 diseases and injuries in 204 countries and territories, 1990–2019: a systematic analysis for the global burden of disease study 2019. Lancet. (2020) 396:1204–22. 10.1016/S0140-6736(20)30925-9 33069326 PMC7567026

[13] Orsini F D'Ambrosio F Scardigno A Ricciardi R Calabrò GE. Epidemiological impact of metabolic syndrome in overweight and obese European children and adolescents: a systematic literature review. Nutrients. (2023) 15:3895. 10.3390/nu15183895 37764679 PMC10536523

[14] Nanditha A Susairaj P Satheesh K Raghavan A Snehalatha C Ramachandran A. The rising prevalence of type 2 diabetes among the youth in southern India-an ancillary analysis of the secular TRends in DiabEtes in India (STRiDE-I) study. J Diabetes. (2024) 16:e13576. 10.1111/1753-0407.13576 38923743 PMC11200006

[15] Cockerham WC Bauldry S Hamby BW Shikany JM Bae S. A comparison of black and white racial differences in health lifestyles and cardiovascular disease. Am J Prev Med. (2017) 52:S56–62. 10.1016/j.amepre.2016.09.019 27989294 PMC5396536

[16] Zhou H Ding X Lan Y Chen S Wu S Wu D. Multi-trajectories of triglyceride-glucose index and lifestyle with cardiovascular disease: a cohort study. Cardiovasc Diabetol. (2023) 22:341. 10.1186/s12933-023-02076-z 38093279 PMC10720233

[17] Noonan JA. A history of pediatric specialties: the development of pediatric cardiology. Pediatr. Res. (2004) 56:298–306. 10.1203/01.PDR.0000132662.73362.96 15181186

[18] Cheng TL Shilkofski N, Pediatric Policy Council. Global child health: beyond surviving to thriving. Pediatr Res. (2019) 86:683–4. 10.1038/s41390-019-0574-6 31499512

[19] Mash R Howe A Olayemi O Makwero M Ray S Zerihun M Reflections on family medicine and primary healthcare in Sub-saharan Africa. BMJ Glob Health. (2018) 3:e000662. 10.1136/bmjgh-2017-000662 29765778 PMC5950631

[20] Melesse DY Mutua MK Choudhury A Wado YD Faye CM Neal S Adolescent sexual and reproductive health in Sub-saharan Africa: who is left behind. BMJ Glob Health. (2020) 5:e002231. 10.1136/bmjgh-2019-002231 32133182 PMC7042602

[21] GBD 2019 Universal Health Coverage Collaborators. Measuring universal health coverage based on an index of effective coverage of health services in 204 countries and territories, 1990–2019: a systematic analysis for the global burden of disease study 2019. Lancet. (2020) 396:1250–84. 10.1016/S0140-6736(20)30750-9 32861314 PMC7562819

[22] Mwatondo A Muturi M Akoko J Nyamota R Nthiwa D Maina J Seroprevalence and related risk factors of Brucella spp. In livestock and humans in garbatula subcounty, isiolo county, Kenya. PLoS Negl Trop Dis. (2023) 17:e0011682. 10.1371/journal.pntd.0011682 37844102 PMC10602376

[23] Huang C Barnett AG Wang X Tong S. Effects of extreme temperatures on years of life lost for cardiovascular deaths: a time series study in Brisbane, Australia. Circ Cardiovasc Qual Outcomes. (2012) 5:609–14. 10.1161/CIRCOUTCOMES.112.965707 22991346

[24] Al-Kindi S Motairek I Khraishah H Rajagopalan S. Cardiovascular disease burden attributable to non-optimal temperature: analysis of the 1990–2019 global burden of disease. Eur J Prev Cardiol. (2023) 30:1623–31. 10.1093/eurjpc/zwad130 37115593

