# Supplementary material for: Global, regional, and national burden of childhood cardiovascular disease: trends from 1990 to 2021
Source: Front Pediatr. 2024 Dec 11;12:1495238. doi: 10.3389/fped.2024.1495238 (PMC11668807; doi:10.3389/fped.2024.1495238)
Supplement: Supplementary file 1 [file Datasheet1.doc]

**Supplementary material:**

**Supplementary Figure**:


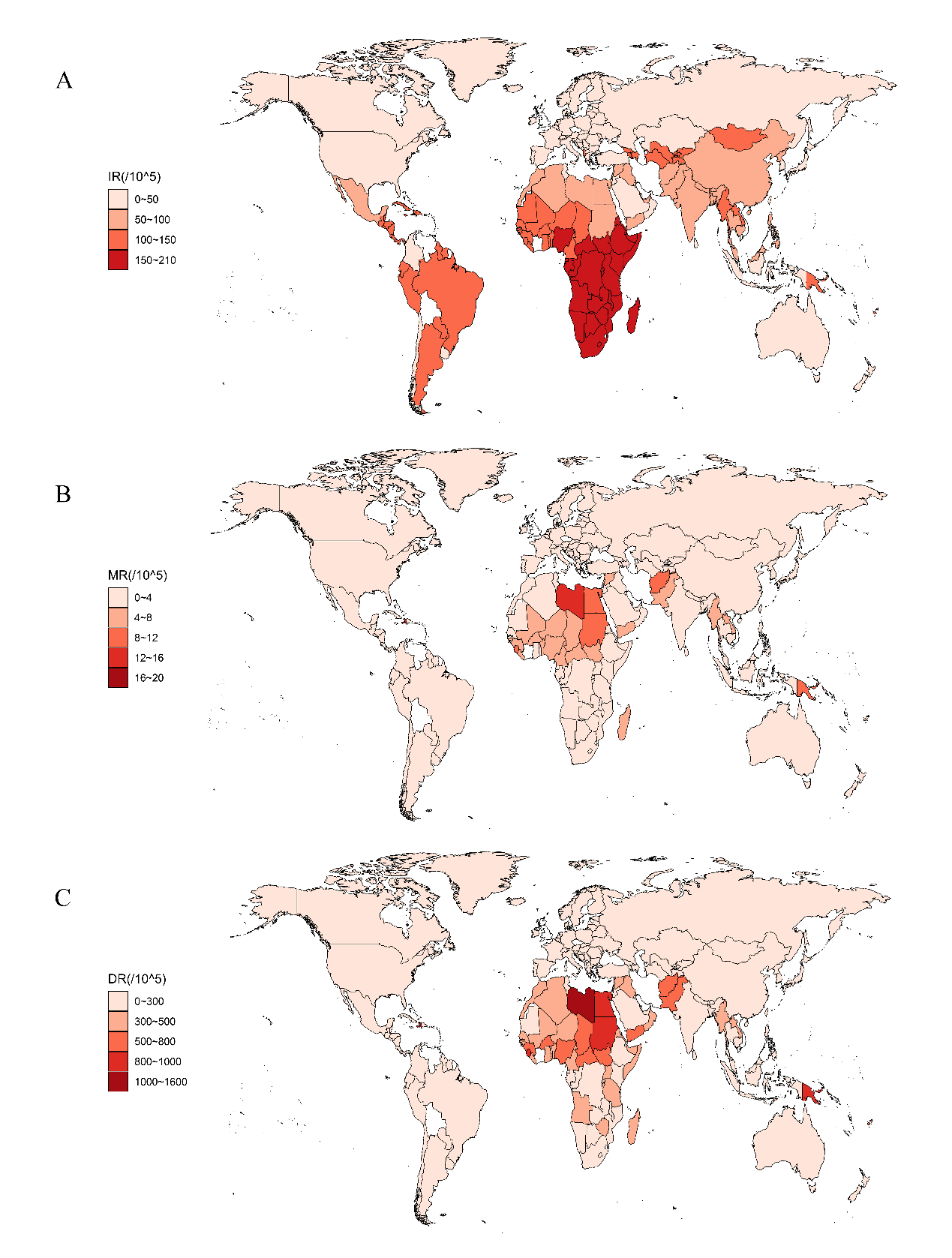


**Supplementary Figure 1**. The incidence, deaths and DALYs rates of diabetes in children in 204 countries and territories. (A) Incidence; (B) Deaths; (C) DALYs.


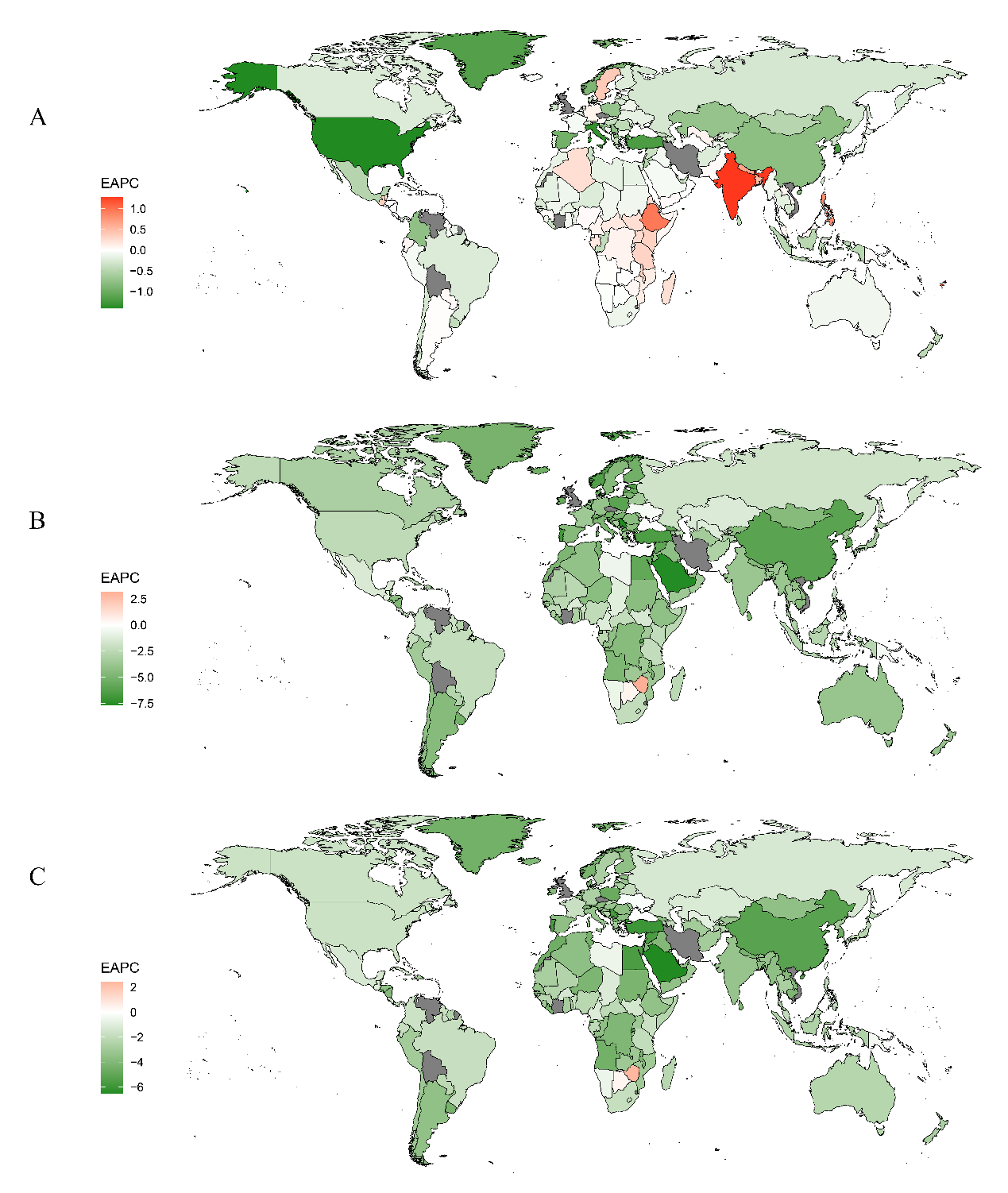


**Supplementary Figure 2**. The national burden of diabetes in children in 204 countries and territories. (A) EAPC for incidence rate. (B) EAPC for deaths rate. (C) EAPC for DALYs rate. EAPC=estimated annual percentage change.

**Supplementary Table**:

Supplementary Table 1. Incidence of Cardiovascular diseases in 0-14 years at the global and regional level

| Rate per 100 000 (95% UI) | | | | | | |
| --- | --- | --- | --- | --- | --- | --- |
|  | 1990 | | 2021 | | 1990-2021 | |
| location | Incidence cases | IR | Incidence cases | IR | Cases change | EAPC |
| Andean Latin America | 17976.63 (12644.88 to 24572.55) | 121.04 (85.14 to 165.45) | 21839.81 (15102.53 to 30297.91) | 120.7 (83.46 to 167.44) | 0.21 (0.17 to 0.26) | -0.04 (-0.05 to -0.02) |
| Australasia | 854.97 (674.51 to 1096.19) | 18.64 (14.71 to 23.9) | 1017.58 (810.98 to 1266.35) | 17.76 (14.15 to 22.1) | 0.19 (0.1 to 0.25) | -0.18 (-0.23 to -0.13) |
| Caribbean | 13800.1 (10002.98 to 18610.26) | 120.92 (87.65 to 163.07) | 14694.1 (10403.54 to 20041.49) | 127.72 (90.43 to 174.2) | 0.06 (0.02 to 0.11) | 0.12 (0.1 to 0.14) |
| Central Asia | 23213.82 (16735.36 to 31513.79) | 92.89 (66.96 to 126.1) | 26172.18 (18654.52 to 35791.03) | 94.57 (67.4 to 129.32) | 0.13 (0.09 to 0.16) | -0.13 (-0.27 to 0) |
| Central Europe | 8039.08 (6514.05 to 9928.54) | 27.27 (22.09 to 33.67) | 4101.99 (3287.86 to 5145.3) | 23.17 (18.57 to 29.07) | -0.49 (-0.51 to -0.47) | -0.7 (-0.77 to -0.64) |
| Central Latin America | 44386.16 (34124.15 to 57404.04) | 68.94 (53 to 89.16) | 42503.14 (31768.21 to 56232.42) | 66.95 (50.04 to 88.58) | -0.04 (-0.08 to -0.01) | -0.18 (-0.22 to -0.14) |
| Central Sub-Saharan Africa | 47440.04 (33381.3 to 66257.88) | 187.52 (131.95 to 261.9) | 114437.34 (78009.84 to 162311.23) | 195.01 (132.94 to 276.6) | 1.41 (1.28 to 1.53) | 0.04 (0.01 to 0.08) |
| East Asia | 381845.53 (293719.81 to 491472.85) | 115.77 (89.05 to 149.01) | 234379.03 (171543.69 to 311139.53) | 87.67 (64.16 to 116.38) | -0.39 (-0.42 to -0.36) | -0.77 (-0.88 to -0.65) |
| Eastern Europe | 12641.98 (9940.79 to 16331.7) | 24.57 (19.32 to 31.74) | 8377.72 (6606.65 to 10689.19) | 23.64 (18.64 to 30.16) | -0.34 (-0.36 to -0.31) | -0.24 (-0.29 to -0.19) |
| Eastern Sub-Saharan Africa | 141903.57 (98973.46 to 193835.2) | 156.68 (109.28 to 214.02) | 310146.41 (208445.72 to 432494.39) | 173.82 (116.82 to 242.39) | 1.19 (1.09 to 1.26) | 0.42 (0.39 to 0.45) |
| Global | 1486136.37 (1115077.02 to 1959529.28) | 85.45 (64.12 to 112.67) | 1861693.2 (1335751.17 to 2531859.51) | 92.54 (66.39 to 125.85) | 0.25 (0.18 to 0.31) | 0.43 (0.36 to 0.5) |
| High-income Asia Pacific | 10507.2 (8313.06 to 13327.57) | 29.85 (23.62 to 37.86) | 6025.49 (4670.46 to 7771.88) | 26.87 (20.83 to 34.66) | -0.43 (-0.45 to -0.41) | -0.52 (-0.59 to -0.45) |
| High-income North America | 19387.22 (15289.75 to 24807.47) | 31.43 (24.79 to 40.22) | 17255.28 (13809.7 to 21799.07) | 26.3 (21.05 to 33.22) | -0.11 (-0.16 to -0.06) | -1.31 (-1.63 to -0.99) |
| High-middle SDI | 170106.22 (139292.33 to 208137.77) | 62.17 (50.91 to 76.07) | 112339.58 (88791.33 to 141452.9) | 48.65 (38.46 to 61.26) | -0.34 (-0.37 to -0.32) | -0.83 (-0.9 to -0.77) |
| High SDI | 55653.7 (45547.02 to 69700.17) | 29.95 (24.51 to 37.51) | 45330.88 (36999.73 to 56659.82) | 26.27 (21.44 to 32.84) | -0.19 (-0.21 to -0.17) | -0.77 (-0.93 to -0.61) |
| Low-middle SDI | 402697.26 (292397.76 to 543353.12) | 85.3 (61.93 to 115.09) | 561620.15 (394482.23 to 773725.05) | 96.86 (68.03 to 133.44) | 0.39 (0.34 to 0.44) | 0.66 (0.56 to 0.77) |
| Low SDI | 285675.75 (205797.27 to 385543.72) | 124.8 (89.9 to 168.42) | 636597.86 (442425.18 to 876581.56) | 138.32 (96.13 to 190.47) | 1.23 (1.14 to 1.3) | 0.45 (0.41 to 0.49) |
| Middle SDI | 570854.17 (425900.9 to 754006.64) | 98.9 (73.79 to 130.63) | 504378.22 (362860.42 to 681816.41) | 88.98 (64.01 to 120.28) | -0.12 (-0.16 to -0.09) | -0.22 (-0.3 to -0.14) |
| North Africa and Middle East | 100523.58 (79271.48 to 127757.01) | 71.55 (56.43 to 90.94) | 129603.07 (96930.06 to 169609.64) | 70.7 (52.87 to 92.52) | 0.29 (0.22 to 0.36) | -0.12 (-0.19 to -0.06) |
| Oceania | 3548.59 (2674.05 to 4636.17) | 132.42 (99.78 to 173) | 7006.43 (5255.35 to 9208.76) | 137.9 (103.43 to 181.24) | 0.97 (0.9 to 1.08) | 0.13 (0.06 to 0.19) |
| South Asia | 288746.73 (205606.03 to 389934.21) | 66.63 (47.44 to 89.98) | 372928.59 (253625.61 to 517464.47) | 73.55 (50.02 to 102.06) | 0.29 (0.22 to 0.35) | 1.04 (0.75 to 1.32) |
| Southeast Asia | 104709.38 (82130.14 to 130872.91) | 61.32 (48.1 to 76.65) | 105044.87 (80757.2 to 135034.52) | 60.84 (46.77 to 78.21) | 0 (-0.04 to 0.04) | 0.02 (-0.06 to 0.09) |
| Southern Latin America | 12278.92 (9057.55 to 16401.58) | 82.26 (60.68 to 109.88) | 12501.01 (8972.19 to 17027) | 86.24 (61.9 to 117.46) | 0.02 (-0.04 to 0.08) | 0.15 (0.12 to 0.19) |
| Southern Sub-Saharan Africa | 35021.72 (23930.65 to 48797.16) | 169.27 (115.67 to 235.86) | 41530.23 (27947.42 to 58351.13) | 172.57 (116.13 to 242.46) | 0.19 (0.15 to 0.22) | -0.07 (-0.13 to -0.02) |
| Tropical Latin America | 76214.4 (52522.92 to 105972.55) | 142.15 (97.96 to 197.66) | 67665.48 (46304.75 to 93866.54) | 134.81 (92.25 to 187.01) | -0.11 (-0.13 to -0.09) | -0.24 (-0.27 to -0.21) |
| Western Europe | 17006.42 (13401.24 to 21542.73) | 23.95 (18.87 to 30.33) | 14742.29 (11732.86 to 18358.79) | 21.64 (17.22 to 26.95) | -0.13 (-0.16 to -0.1) | -0.45 (-0.55 to -0.36) |
| Western Sub-Saharan Africa | 126090.34 (95637.02 to 165246.14) | 143.48 (108.83 to 188.04) | 309721.18 (226562.54 to 418223.69) | 144.21 (105.49 to 194.74) | 1.46 (1.35 to 1.54) | -0.05 (-0.07 to -0.03) |

***Supplementary Table*** 2.Deaths of Cardiovascular diseases in 0-14 years at the global and regional level

| Rate per 100 000 (95% UI) | | | | | | |
| --- | --- | --- | --- | --- | --- | --- |
|  | 1990 | | 2021 | | 1990-2021 | |
| location | Deaths cases | MR | Deaths cases | MR | Cases change | EAPC |
| Andean Latin America | 904.59 (802.82 to 1061.13) | 6.09 (5.41 to 7.14) | 329.08 (271.24 to 401.63) | 1.82 (1.5 to 2.22) | -0.64 (-0.72 to -0.54) | -3.42 (-3.57 to -3.26) |
| Australasia | 62.51 (59.53 to 65.43) | 1.36 (1.3 to 1.43) | 19.83 (17.43 to 22.64) | 0.35 (0.3 to 0.4) | -0.68 (-0.72 to -0.63) | -3.87 (-4.08 to -3.66) |
| Caribbean | 1104.3 (932.68 to 1300.78) | 9.68 (8.17 to 11.4) | 696.63 (532.26 to 872.33) | 6.05 (4.63 to 7.58) | -0.37 (-0.52 to -0.19) | -0.99 (-1.3 to -0.68) |
| Central Asia | 483.81 (447.83 to 526.48) | 1.94 (1.79 to 2.11) | 252.61 (216.16 to 294.56) | 0.91 (0.78 to 1.06) | -0.48 (-0.55 to -0.38) | -2.25 (-2.54 to -1.95) |
| Central Europe | 671.88 (642.46 to 709.6) | 2.28 (2.18 to 2.41) | 74.49 (65.23 to 83.03) | 0.42 (0.37 to 0.47) | -0.89 (-0.9 to -0.87) | -5.08 (-5.25 to -4.9) |
| Central Latin America | 2059.36 (1941.09 to 2197.85) | 3.2 (3.01 to 3.41) | 930.16 (752.05 to 1150.97) | 1.47 (1.18 to 1.81) | -0.55 (-0.64 to -0.44) | -1.78 (-2.15 to -1.4) |
| Central Sub-Saharan Africa | 3122.49 (2201.81 to 4209.9) | 12.34 (8.7 to 16.64) | 1707.21 (1152.82 to 2256.09) | 2.91 (1.96 to 3.84) | -0.45 (-0.56 to -0.31) | -4.35 (-4.63 to -4.08) |
| East Asia | 20490.11 (17761 to 23580.08) | 6.21 (5.38 to 7.15) | 2336.84 (1926.27 to 2748.04) | 0.87 (0.72 to 1.03) | -0.89 (-0.91 to -0.86) | -5.98 (-6.17 to -5.78) |
| Eastern Europe | 514.77 (498.04 to 532.11) | 1 (0.97 to 1.03) | 174.91 (164.22 to 183.6) | 0.49 (0.46 to 0.52) | -0.66 (-0.68 to -0.64) | -1.72 (-2.36 to -1.07) |
| Eastern Sub-Saharan Africa | 6623.01 (5408.93 to 8284.08) | 7.31 (5.97 to 9.15) | 4597.12 (3608.44 to 5605.5) | 2.58 (2.02 to 3.14) | -0.31 (-0.46 to -0.14) | -3.23 (-3.31 to -3.15) |
| Global | 126621.81 (110313.33 to 151396.47) | 7.28 (6.34 to 8.71) | 51727.58 (43452.28 to 60331.54) | 2.57 (2.16 to 3) | -0.59 (-0.65 to -0.52) | -2.97 (-3.09 to -2.85) |
| High-income Asia Pacific | 681.48 (610.01 to 759.66) | 1.94 (1.73 to 2.16) | 113.89 (104.98 to 121.65) | 0.51 (0.47 to 0.54) | -0.83 (-0.85 to -0.81) | -4.36 (-4.51 to -4.2) |
| High-income North America | 1307.58 (1280.93 to 1334.76) | 2.12 (2.08 to 2.16) | 672.59 (609.91 to 734.74) | 1.02 (0.93 to 1.12) | -0.49 (-0.54 to -0.44) | -2.46 (-2.6 to -2.32) |
| High-middle SDI | 12650.06 (11220.47 to 14646.67) | 4.62 (4.1 to 5.35) | 2135.19 (1873.5 to 2408.38) | 0.92 (0.81 to 1.04) | -0.83 (-0.86 to -0.8) | -4.83 (-5.02 to -4.65) |
| High SDI | 3999.93 (3766.82 to 4285.45) | 2.15 (2.03 to 2.31) | 1168.05 (1070.45 to 1243.72) | 0.68 (0.62 to 0.72) | -0.71 (-0.74 to -0.69) | -3.62 (-3.69 to -3.55) |
| Low-middle SDI | 50190.96 (43162.39 to 60386.14) | 10.63 (9.14 to 12.79) | 19572.62 (16420.4 to 23041.49) | 3.38 (2.83 to 3.97) | -0.61 (-0.68 to -0.53) | -3.25 (-3.37 to -3.12) |
| Low SDI | 24850.32 (20138.14 to 32407.72) | 10.86 (8.8 to 14.16) | 19854.15 (15376.15 to 24683.91) | 4.31 (3.34 to 5.36) | -0.2 (-0.35 to -0.01) | -2.85 (-2.95 to -2.76) |
| Middle SDI | 34826.38 (31354.63 to 40043.94) | 6.03 (5.43 to 6.94) | 8934.2 (7856.83 to 10122.38) | 1.58 (1.39 to 1.79) | -0.74 (-0.79 to -0.7) | -3.79 (-3.98 to -3.6) |
| North Africa and Middle East | 34760.4 (29684.32 to 42203.61) | 24.74 (21.13 to 30.04) | 9616.68 (7978.6 to 11568.54) | 5.25 (4.35 to 6.31) | -0.72 (-0.78 to -0.66) | -4.39 (-4.57 to -4.22) |
| Oceania | 266.09 (193.78 to 357.6) | 9.93 (7.23 to 13.34) | 446.17 (340.16 to 565.67) | 8.78 (6.69 to 11.13) | 0.68 (0.29 to 1.2) | -0.21 (-0.52 to 0.1) |
| South Asia | 28855.19 (23349.76 to 36145.23) | 6.66 (5.39 to 8.34) | 12569.56 (10320.27 to 15016.94) | 2.48 (2.04 to 2.96) | -0.56 (-0.65 to -0.46) | -2.85 (-3 to -2.71) |
| Southeast Asia | 9676.19 (8317.73 to 11814.26) | 5.67 (4.87 to 6.92) | 3828.65 (3263.28 to 4445.83) | 2.22 (1.89 to 2.58) | -0.6 (-0.69 to -0.51) | -2.8 (-2.86 to -2.74) |
| Southern Latin America | 562.76 (538.06 to 587.32) | 3.77 (3.6 to 3.93) | 113.5 (98.67 to 129.3) | 0.78 (0.68 to 0.89) | -0.8 (-0.82 to -0.77) | -4.44 (-4.77 to -4.12) |
| Southern Sub-Saharan Africa | 709.82 (608.27 to 845.84) | 3.43 (2.94 to 4.09) | 571.68 (473.55 to 687.17) | 2.38 (1.97 to 2.86) | -0.19 (-0.34 to -0.02) | -0.73 (-1.07 to -0.39) |
| Tropical Latin America | 1883.2 (1693.63 to 2092.02) | 3.51 (3.16 to 3.9) | 716.49 (594.19 to 850.97) | 1.43 (1.18 to 1.7) | -0.62 (-0.69 to -0.54) | -2.24 (-2.61 to -1.86) |
| Western Europe | 1082.24 (1060.97 to 1104.92) | 1.52 (1.49 to 1.56) | 241.44 (219.68 to 260.62) | 0.35 (0.32 to 0.38) | -0.78 (-0.8 to -0.76) | -4.57 (-4.67 to -4.48) |
| Western Sub-Saharan Africa | 10800.04 (8614.49 to 13754.26) | 12.29 (9.8 to 15.65) | 11718.04 (8337.5 to 15045.83) | 5.46 (3.88 to 7.01) | 0.09 (-0.13 to 0.37) | -2.43 (-2.59 to -2.27) |

Supplementary Table 3.DALYs of Cardiovascular diseases in 0-14 years at the global and regional level

| Rate per 100 000 (95% UI) | | | | | | |
| --- | --- | --- | --- | --- | --- | --- |
|  | 1990 | | 2021 | | 1990-2021 | |
| location | DALYs cases | DR | DALYs cases | DR | Cases change | EAPC |
| Andean Latin America | 84835.7 (75641.79 to 99113.05) | 571.21 (509.3 to 667.34) | 36344.34 (30684.41 to 44161.56) | 200.86 (169.58 to 244.06) | -0.57 (-0.66 to -0.48) | -2.98 (-3.12 to -2.84) |
| Australasia | 6702.69 (6221.8 to 7347.03) | 146.16 (135.67 to 160.21) | 3531.56 (2881.55 to 4307.84) | 61.62 (50.28 to 75.17) | -0.47 (-0.54 to -0.4) | -2.41 (-2.55 to -2.28) |
| Caribbean | 100861.78 (86076.63 to 117607.71) | 883.79 (754.24 to 1030.52) | 65678.42 (51101.49 to 81451.29) | 570.86 (444.16 to 707.96) | -0.35 (-0.49 to -0.18) | -0.92 (-1.21 to -0.63) |
| Central Asia | 51609.32 (47203.34 to 57314.1) | 206.51 (188.88 to 229.34) | 32000.99 (27281.82 to 37801.03) | 115.63 (98.58 to 136.59) | -0.38 (-0.46 to -0.3) | -1.79 (-2.01 to -1.57) |
| Central Europe | 68716.6 (64595.08 to 73843.21) | 233.07 (219.09 to 250.46) | 11501.98 (9775.71 to 13687.83) | 64.98 (55.23 to 77.33) | -0.83 (-0.85 to -0.81) | -3.97 (-4.06 to -3.89) |
| Central Latin America | 200061.59 (187015.36 to 215854.47) | 310.75 (290.48 to 335.28) | 100065.71 (83227.89 to 122006.28) | 157.62 (131.1 to 192.18) | -0.5 (-0.58 to -0.4) | -1.58 (-1.91 to -1.26) |
| Central Sub-Saharan Africa | 288125.49 (208588.75 to 384747.59) | 1138.9 (824.51 to 1520.82) | 182857.13 (133488.62 to 235131.11) | 311.61 (227.48 to 400.69) | -0.37 (-0.49 to -0.21) | -3.97 (-4.19 to -3.75) |
| East Asia | 1927410.41 (1682958.9 to 2188361.64) | 584.36 (510.24 to 663.47) | 300387.37 (250636.83 to 353125.42) | 112.36 (93.75 to 132.08) | -0.84 (-0.87 to -0.81) | -5.16 (-5.27 to -5.05) |
| Eastern Europe | 57231.4 (52821.26 to 62041.41) | 111.21 (102.64 to 120.56) | 25353.08 (21798.4 to 29731.95) | 71.53 (61.5 to 83.88) | -0.56 (-0.59 to -0.52) | -1.13 (-1.55 to -0.7) |
| Eastern Sub-Saharan Africa | 629804.13 (521378.85 to 779640.47) | 695.37 (575.66 to 860.81) | 506770.52 (416576.2 to 614723.36) | 284.01 (233.47 to 344.52) | -0.2 (-0.37 to -0.02) | -2.8 (-2.87 to -2.73) |
| Global | 11664257.27 (10332913.39 to 13769950.1) | 670.69 (594.14 to 791.76) | 5300049.98 (4579016.42 to 6083865.89) | 263.44 (227.6 to 302.4) | -0.55 (-0.61 to -0.48) | -2.68 (-2.78 to -2.58) |
| High-income Asia Pacific | 68439.27 (61347.77 to 76002.13) | 194.43 (174.29 to 215.92) | 16924.27 (14357.24 to 19821.22) | 75.47 (64.02 to 88.39) | -0.75 (-0.79 to -0.72) | -3.18 (-3.28 to -3.07) |
| High-income North America | 144963.37 (134958.69 to 157159.88) | 235.03 (218.81 to 254.81) | 95207.74 (81335.03 to 112930.96) | 145.09 (123.95 to 172.1) | -0.34 (-0.4 to -0.28) | -1.65 (-1.75 to -1.56) |
| High-middle SDI | 1197550.33 (1068222.55 to 1378383.48) | 437.66 (390.4 to 503.75) | 261370.01 (226811.14 to 299504.09) | 113.2 (98.23 to 129.72) | -0.78 (-0.82 to -0.74) | -4.15 (-4.26 to -4.05) |
| High SDI | 416977.03 (386500.26 to 451116.05) | 224.41 (208.01 to 242.79) | 172966.94 (147717.9 to 204832.92) | 100.25 (85.62 to 118.72) | -0.59 (-0.63 to -0.53) | -2.54 (-2.59 to -2.49) |
| Low-middle SDI | 4515357.04 (3905283.69 to 5388236.32) | 956.42 (827.19 to 1141.3) | 1914956.9 (1633696.89 to 2227863.24) | 330.26 (281.75 to 384.22) | -0.58 (-0.65 to -0.5) | -3.01 (-3.13 to -2.9) |
| Low SDI | 2270874.98 (1852477.04 to 2909438.72) | 992.02 (809.25 to 1270.98) | 1953669.52 (1560954.86 to 2377201.56) | 424.5 (339.17 to 516.53) | -0.14 (-0.29 to 0.03) | -2.63 (-2.71 to -2.55) |
| Middle SDI | 3253873.19 (2954670.18 to 3702133.29) | 563.72 (511.88 to 641.38) | 990953.97 (877181.28 to 1125439.64) | 174.81 (154.74 to 198.54) | -0.7 (-0.75 to -0.65) | -3.35 (-3.5 to -3.2) |
| North Africa and Middle East | 3103369.61 (2668850.32 to 3759109.05) | 2209.03 (1899.73 to 2675.8) | 935427.57 (791552.29 to 1098765.98) | 510.26 (431.78 to 599.36) | -0.7 (-0.76 to -0.63) | -4.17 (-4.33 to -4.01) |
| Oceania | 24329.1 (18206.8 to 32192.09) | 907.85 (679.39 to 1201.26) | 41105.4 (31824.77 to 51578.17) | 809.02 (626.36 to 1015.14) | 0.69 (0.32 to 1.18) | -0.19 (-0.49 to 0.11) |
| South Asia | 2602684.84 (2126414.64 to 3241037.88) | 600.58 (490.68 to 747.88) | 1226609.46 (1028499.63 to 1444475.6) | 241.92 (202.85 to 284.89) | -0.53 (-0.62 to -0.42) | -2.61 (-2.75 to -2.47) |
| Southeast Asia | 883297.15 (766595.44 to 1071682.92) | 517.31 (448.96 to 627.64) | 380631.95 (332942.9 to 437350.89) | 220.46 (192.84 to 253.31) | -0.57 (-0.65 to -0.48) | -2.56 (-2.61 to -2.51) |
| Southern Latin America | 55184.34 (52337.2 to 58601.66) | 369.71 (350.63 to 392.6) | 16295.68 (13791.32 to 19528.99) | 112.42 (95.14 to 134.72) | -0.7 (-0.74 to -0.66) | -3.43 (-3.74 to -3.11) |
| Southern Sub-Saharan Africa | 72113.79 (62338.36 to 84429.31) | 348.56 (301.31 to 408.08) | 61224.44 (51968.46 to 72213.51) | 254.4 (215.94 to 300.07) | -0.15 (-0.28 to -0.01) | -0.64 (-0.92 to -0.36) |
| Tropical Latin America | 187874.24 (170874.47 to 210368.13) | 350.42 (318.71 to 392.38) | 85877.49 (71876.71 to 102872.39) | 171.09 (143.2 to 204.95) | -0.54 (-0.62 to -0.47) | -1.83 (-2.12 to -1.53) |
| Western Europe | 114601.38 (107711.35 to 123862.79) | 161.37 (151.67 to 174.41) | 41885.8 (34154.56 to 51472.91) | 61.49 (50.14 to 75.56) | -0.63 (-0.68 to -0.58) | -3.07 (-3.17 to -2.97) |
| Western Sub-Saharan Africa | 992041.11 (807577.85 to 1247485.05) | 1128.86 (918.96 to 1419.54) | 1134369.08 (856783.92 to 1417839.03) | 528.19 (398.94 to 660.19) | 0.14 (-0.06 to 0.42) | -2.29 (-2.44 to -2.14) |
